# Molecular Mechanisms of Fiber Differential Development between *G. barbadense* and *G. hirsutum* Revealed by Genetical Genomics

**DOI:** 10.1371/journal.pone.0030056

**Published:** 2012-01-11

**Authors:** Xiangdong Chen, Wangzhen Guo, Bingliang Liu, Yuanming Zhang, Xianliang Song, Yu Cheng, Lili Zhang, Tianzhen Zhang

**Affiliations:** National Key Laboratory of Crop Genetics and Germplasm Enhancement, Cotton Research Institute, Nanjing Agricultural University, Nanjing, China; National Taiwan University, Taiwan

## Abstract

Cotton fiber qualities including length, strength and fineness are known to be controlled by genes affecting cell elongation and secondary cell wall (SCW) biosynthesis, but the molecular mechanisms that govern development of fiber traits are largely unknown. Here, we evaluated an interspecific backcrossed population from *G. barbadense* cv. Hai7124 and *G. hirsutum* acc. TM-1 for fiber characteristics in four-year environments under field conditions, and detected 12 quantitative trait loci (QTL) and QTL-by-environment interactions by multi-QTL joint analysis. Further analysis of fiber growth and gene expression between TM-1 and Hai7124 showed greater differences at 10 and 25 days post-anthesis (DPA). In this two period important for fiber performances, we integrated genome-wide expression profiling with linkage analysis using the same genetic materials and identified in total 916 expression QTL (eQTL) significantly (*P*<0.05) affecting the expression of 394 differential genes. Many positional *cis*-/*trans*-acting eQTL and eQTL hotspots were detected across the genome. By comparative mapping of eQTL and fiber QTL, a dataset of candidate genes affecting fiber qualities was generated. Real-time quantitative RT-PCR (qRT-PCR) analysis confirmed the major differential genes regulating fiber cell elongation or SCW synthesis. These data collectively support molecular mechanism for *G. hirsutum* and *G. barbadense* through differential gene regulation causing difference of fiber qualities. The down-regulated expression of abscisic acid (ABA) and ethylene signaling pathway genes and high-level and long-term expression of positive regulators including auxin and cell wall enzyme genes for fiber cell elongation at the fiber developmental transition stage may account for superior fiber qualities.

## Introduction

Cultivated tetraploid cotton yields approximate 97% natural fiber used widely by the textile industry. The two tetraploid species, extra-long staple cotton (*Gossypium barbadense* L.) and Upland cotton (*Gossypium hirsutum* L.) are allotetraploids (2n = 4x = 52) composed of two ancestral genomes designated A-subgenome (At) and D-subgenome (Dt), originating from a polyploidy event ∼1–2 million years ago [1]. The extra-long staple cotton has superior fiber quality properties such as length, strength and fineness, while Upland cotton is characterized by its high yield. Therefore, these two species can be used to dissect the molecular and genetic basis of fiber qualities which will be very important for improved breeding to further meet global demands for cotton.

Cotton fibers are highly elongated single-celled seed trichomes that initiate from the seed coat, and they serve as an experimental model for cell elongation and cellulose synthesis. The final fiber quality results from a complex developmental process, which includes four distinct, but overlapping steps: initiation, elongation, secondary cell wall (SCW) biosynthesis and maturation/dehydration [2]–[7]. Although many studies have been focused on identification of key genes controlling cotton fiber elongation and cell wall biosynthesis [6]–[16], the molecular mechanism of fiber cell elongation is still not fully understood. Integration of plasmodesmatal gating and expression of sucrose and K^+^ transporters and expansin appear to control fiber cell elongation in a coordinate manner [8], while a fiber-specific β-1,3-glucanase gene (*GhGluc1*) also may play a role in fiber elongation by degrading callose, thus opening the plasmodesmata [9]. Previous microarray analysis has indicated that the genes up-regulated during cell elongation are involved in cell wall structure and biogenesis, formation of the cytoskeleton and energy/carbohydrate metabolism, which corresponds with the high rates of cell expansion occurring during fiber elongation [10]. Several auxin and ethylene related genes have been identified as potential regulators of fiber elongation [11]–[14], including 1-Aminocyclopropane-1-Carboxylic acid Oxidase (*ACO*) genes which are responsible for the ethylene promote fiber cell elongation in vitro [14]. A novel mechanism of modulating cell elongation processes by regulating redox levels was uncovered in recent findings, in which three genes *GAST1-like*, *Cop1/BONZAI* and *Pex1*, highly up-regulated in the A-genome long fiber relative to the F-genome short fiber, were found to regulate fiber cell elongation by controlling H_2_O_2_ levels [15]. Although considerable functional genomics work on cotton fiber development has been carried out [10], [13]–[16], these studies have mainly been conducted with the *G. hirsutum* species and derivative fiber mutants. Very little information has been accumulated on the differences in fiber gene expression between different tetraploid cotton species. Recently, only two reports have focused on the transcriptional changes between *G. hirsutum* and *G. barbadense* fibers using microarrays [17]–[18]. However, fiber quality genes are less well-characterized, and little is known about underlying biological causes of these differences in cotton fiber qualities.

With the advance of high throughput gene expression profiling technologies, the concept of “genetical genomics” was proposed [19]. In traditional cotton fiber quality quantitative trait loci (QTL) analyses, linkage mapping leads to the detection of genomic regions which are associated with phenotypic variations in the different interspecific populations [20]–[25]. Genetical genomics employs this same approach, except that the phenotypes are levels in gene expression resulting in the detection of expression QTL (eQTL). Genetical genomics is a new strategy for identifying genes underlying complex phenotypes [19], which can be used to infer the chromosomal positions of thousands of genes and is particularly valuable for species with genomes that are not fully sequenced. This approach has been applied in model plants such as *Arabidopsis* [26]–[28] and rice [29], and to crops including barley [30], maize [31]–[33] and wheat [34]. However, there has been not yet been a report in cotton using this method.

Recent advances in microarray technology applied in cotton have provided a high-throughput platform for measuring fiber gene expression. Here, we present the fiber differential gene expression profile between *Gossypium hirsutum* acc. TM-1 and *Gossypium barbadense* cv. Hai7124. The cotton fiber gene expression profiles were integrated with information on genetic markers at two key developmental stages in the interspecific [(TM-1×Hai7124)×TM-1] BC_1_ segregating population [35], further combining QTL mapped for fiber qualities and eQTL. This novel approach to dissecting the genetic basis of complex fiber traits in cotton will allow in-depth characterization of certain targeted QTL and functional genes underlying fiber quality properties and deepen our understanding of molecular mechanisms controlling fiber qualities.

## Results

### Fiber quality performances and QTL mapping

**Figure 1:**
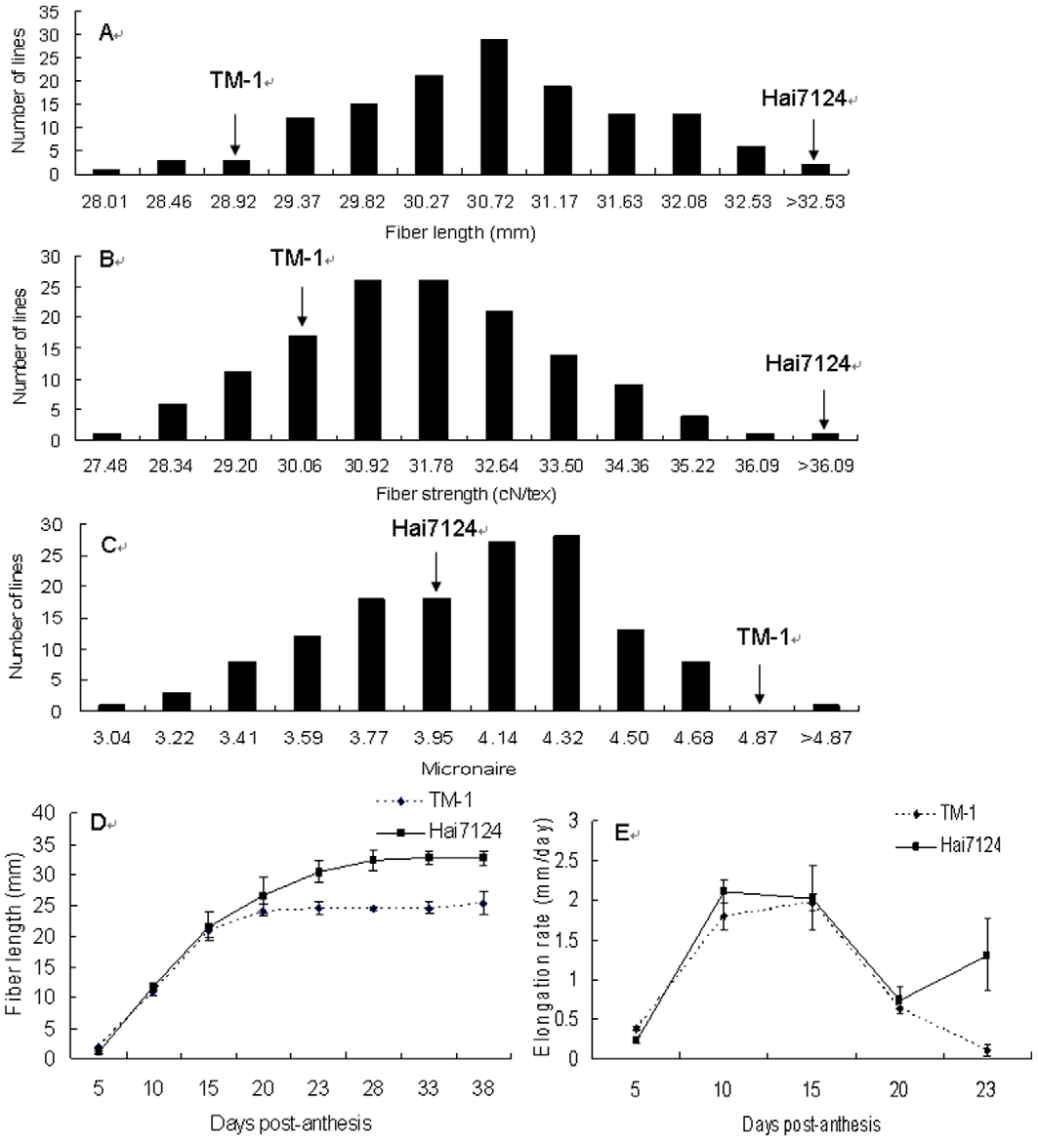
The frequency distribution of fiber quality traits and dynamic change of fiber length during development. The mean values of fiber length (a), strength (b) and micronaire (c) obtained from four-year data of BC_1_S_1_ family lines. Average phenotypic values for parents (*G. hirsutum* acc. TM-1 and *G. barbadense* cv. Hai7124) are indicated by arrows. (x-axis: phenotypic value; y-axis: number of lines). (d). TM-1 and Hai7124 cotton fiber length at different development stages; (e). TM-1 and Hai7124 cotton fiber elongation rate (increasing length per day) at different development stages. Error bars are the standard deviation calculated with seeds from six biological replicates.

Fiber quality data collected from (TM-1×Hai7124) TM-1, an interspecific BC_1_S_1_ mapping population, grown in the field in Nanjing, China in 2003, 2004, 2005 and 2008 confirmed their large variability: mean fiber length (FL) varied from 28 to 33 mm (parents TM-1 and Hai7124 were at 29 and 33 mm on average, respectively), fiber strength (FS) from 27 to 37 g/tex (parents at 30 and 37 g/tex), and fiber micronaire (FM) or fineness values from 3 to 5 (parents at 4.8 and 4.0). There were statistically significant differences between TM-1 and Hai7124 for FL, FS and FM at the *P*<0.01 level. Hai7124 exhibited longer, stronger and finer fiber (lower micronaire values) traits than did TM-1. The fiber quality traits from the combined four-year results exhibited continuous normal distribution in this interspecific BC_1_S_1_ population, typical of quantitative traits (Figure 1a–c).

A total of 12 fiber quality QTL were detected by multi-QTL joint analysis [36], of which eleven were commonly detected by the composite interval mapping (CIM) approach [37] using four-year fiber trait data obtained from interspecific backcrossed population (Table 1). A strong QTL for FL (*qFL-A11-2*) with the highest logarithm of odds (LOD) score was mapped on chromosome A11 (chr. A11), and the alleles from the superior parent Hai7124 at this QTL increased FL. Three QTL for FS were found on chr. A3 and A5; the positive alleles at *qFS-A3-1* and *qFS-A3-2* were from TM-1, whereas the positive allele at *qFS-A5* came from Hai7124. Two major QTL *qFM-D1* and *qFM-A9* were also detected at the significance level of *P*<0.05. At the two QTL for fiber fineness, the Hai7124 allele conferred the finer fiber trait (lower micronaire value). In addition, six QTL-by-environment interactions were tagged (Table 1). The QTL for FL and FS were enriched on regions of chr. A3, which contained three QTL. This suggests that pleiotropic effects and/or close linkage may exist in the same region of QTL for fiber qualities.

**Table 1:** Biometrical parameters of QTL (LOD≥3) affecting quality traits of cotton fiber.

			Multi-QTL joint analysis				Composite interval mapping				
	Traits	QTL	Pos.	LOD	Additive^a^ (a1;a2;a3;a4)	Involving markers	Years	Pos.(cM)	LOD	Additive	R^2^(%)^c^
Major	FL	*qFL-A11-2*	126	7.9	0.42	BNL2589-330	04,05,08^b^	122.5–125.6	4.5–8.4*	0.95–1.51	11–21
	FS	*qFS-A3-1*	63.5	3.2	−0.47	NAU1167-200	03^b^	63.5	3.5*	−1.55	10
		*qFS-A3-2*	78	3.5	−0.48	NAU3083-175	03	78	4.1*	−1.64	11
		*qFS-A5*	75.3	3.6	0.51	NAU779-380	05^b^	75	9.1*	3.74	27
	FM	*qFM-D1*	74.9	4	−0.11	BNL2646-125					
		*qFM-A9*	135	3.5	−0.1	BNL4053-205	05^b^	132.8	10.6*	−0.43	27
G×E	FL	*qFL-A1*	55.8	4.7	−0.10;0.00;−0.42;0.52	BNL1355-370	05	47.3	3.3	−0.92	8
		*qFL-A3*	58.7	5	−0.50;0.27;−0.09;0.32	NAU5444-280	03	63.5	4.7*	−1.19	13
		*qFL-A5*	12.1	3.7	−0.14;−0.03;0.45;−0.28	NAU3607-200	05	12.1	4.6*	1.06	10
		*qFL-A11-1*	118	3.2	−0.47;−0.06;0.10;0.43	BNL1151-170	05,08^b^	113.6–118.2	2.9–6.4*	1.08–1.31	11–16
	FM	*qFM-D4*	27.3	3.6	0.05;0.13;0.01;−0.18	JESPR65-120	04^b^	27.3	2.76	1.25	7
		*qFM-D12*	62.9	3.9	−0.05;−0.05;−0.10;0.21	Y2583	08^b^	62.9	3.59*	0.78	14

G×E indicates genotype×environment interactions. FL, FS, and FM were shortening from fiber length, strength, and micronaire.

a Additive effects of QTL-by-environment interaction in each year; a1;a2;a3;a4 represent four year environment interaction additive effects; a positive value indicates the genotype from the parent Hai124 toward increase the value, a negative value indicates the genotype from the TM-1 toward increase the trait value.

b The QTL in each year calculated from increased 2147 markers with 0.5-cM intervals throughput for linkage mapping results.

c R^2^ represents percentage phenotypic variation explained; *Significant QTL by permutations (p<0.05).

### Kinetics of fiber growth and interspecific and temporal variations of gene expression in the different species

To reveal the differential feature of fiber development that may account for fiber qualities, we measured dynamic changes in FL of TM-1 and Hai7124 at different time points (5–38 days post-anthesis, DPA) and observed that the length increased approximately linearly over the first 15–20 DPA in TM-1 and 23–28 DPA in Hai7124 (Figure 1d). Under these growth conditions the maximum elongation rate occurred at 10–15 DPA and decreased abruptly from 15 DPA, but Hai7124 fiber maintained a higher elongation rate than TM-1 between 20–23 DPA (Figure 1e). Finally fiber elongation in TM-1 ceased before 23 DPA, but in Hai7124 lasted until ∼33 DPA. Thus, further analyses of transcript accumulation were required to determine the cause of these differences in TM-1 and Hai7124 fiber development.

**Figure 2:**
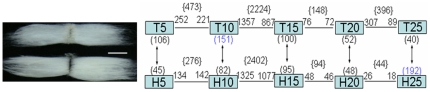
Number of differentially expression genes from interspecific and temporal comparison. A representative image of mature single cell seed trichomes (“cotton fiber”) from cultivar *Gossypium hirsutum* acc. TM-1 (up) and *Gossypium barbadense* cv. Hai7124 (down) (bar = 9.5 mm). Microarray analysis was performed for three replicates of each of five stages (represented by boxes) for both accessions. T5-T25 and H5-H25 were indicated microarray samples for TM-1 and Hai7124 at 5, 10, 15, 20, and 25 DPA, respectively. Numbers marked on the line designate the number of genes up-regulated at least 2-fold (FDR<0.01) relative to their adjacent developmental stage, with the total given in brackets {}. Numbers marked in brackets ( ) denote the numbers of differentially expression genes between TM-1 and Hai7124 at each stage.

To investigate differentially regulated genes in the fiber developmental process, we hybridized a cotton fiber cDNA microarray (GPL2610) with RNA samples from TM-1 and Hai7124 fiber at five different developmental time points, 5, 10, 15, 20 and 25 DPA. In all, 15 two-channel slides were carried out using paired cDNA samples from TM-1 and Hai7124 fibers with three replicates at each stage. We extracted the single channel intensity instead of the ratio value from each two-channel hybridization for further analysis, a strategy that was previously reported to be more flexible and valid [38]–[40]. We compared mRNA expression levels in developing fiber cells between adjacent time points within and between species using the empirical Bayes method in the LIMMA software [41] based on the criterion of false discovery rate (FDR)<0.05 and fold change (FC)≥2 (Figure 2). Within each species, the number of differentially expressed genes (DEGs) was unequally distributed between adjacent time points. The expression changes were biased toward the mid-stage (10–15 DPA), the transition stage from fiber elongation to SCW biosynthesis, with 2,224 (68.6%) and 2,402 (85.3%) differential genes in TM-1 and Hai7124, respectively. Compared with Hai7124, expression changes in TM-1 were biased toward early and later stages of development. In Hai7124, 276 (5 vs. 10 DPA), 94 (15 vs. 20 DPA) and 44 (20 vs. 25 DPA) genes were differentially expressed, whereas in TM-1, 473, 148 and 396 genes were differentially expressed at the respective time points (Fig. 2). When comparing both species at each stage, a total of 106 and 151 genes were up-regulated by 2 fold in TM-1 at 5 and 10 DPA, respectively, while lower numbers (45 and 82) were observed in Hai7124. Similar numbers of up-regulated genes in TM-1 and Hai7124 were found at 15 or 20 DPA. Most noteworthy is the observation that at 25 DPA, a much larger number (232 total) of genes were differentially expressed between TM-1 and Hai7124, with a bias toward up-regulation in Hai7124 (192 in Hai7124 vs. 40 in TM-1). Most of the 192 genes were preferentially expressed during the fiber fast elongation stage. Some among these genes have previously been described to regulate fiber elongation [14], such as genes encoding cell wall-loosening enzymes expansin (*EXP1*) and pectin methylesterase (*PME*); cell cytoskeleton genes tubulin beta-1 (*TUB1*) and profilin; and antiauxin-resistant 3 (*AAR3*). These genes were more highly expressed in *G. barbadense* than in *G. hirsutum* at 25 DPA (Figure S1). This observation was consistent with elongation of Hai7124 fibers which lasted until ∼33 DPA, but only until ∼23 DPA in TM-1 (Figure 1d). These results suggest long-term expression of these fiber elongation genes in Hai7124, whereas expression of these genes decreases rapidly at 20–25 DPA in TM-1. Thus, the two periods at 10 and 25 DPA with higher numbers of DEGs were determined to be crucial for identification of fiber genes regulating elongation at the fast elongation stage and determination of *G. barbadense* fiber continuing elongation or the termination of *G. hirsutum* fiber elongation at the transition stage.

### Genome-wide linkage analysis of expression profiles

We extensively investigated gene expression in fiber cells of 66 randomly selected BC_1_S_1_ lines as well as the two parents TM-1 and Hai7124 using another cDNA microarray platform (GPL8569) with abundant probes. A total of 13,760 and 13,411 active transcripts at 10 and 25 DPA, respectively, were detected and 142 and 302 differentially expressed genes (DEGs) between TM-1 and Hai7124 further identified (Table S1). Furthermore, 444 gene expression traits in 66 cotton BC_1_S_1_ lines were used to conduct eQTL analysis. Out of these 444 DEGs, 394 genes (89%) were detected to have 916 significant eQTL (*P*<0.05), including 293 and 623 eQTL at 10 and 25 DPA, respectively, by linkage analysis (Table 2, Table S2). Over a fourth of these genes had only one identified eQTL, and the remaining three fourths had two to seven. The median explained variance of expression (R^2^ values) across all 916 eQTL was 17% with a range from 8% to nearly 43.8%. We found that 71% of the eQTL individually explained less than 20% of the variations in expression, and 29% of the eQTL explained higher variations for the corresponding genes. Overall, these eQTLs were similarly distributed in the A-subgenome (At) and D-subgenome (Dt), although eQTL seemed to favor Dt (130 in At vs. 163 in Dt) at 10 DPA, while they tended to be on At (364 in At vs. 259 in Dt) at 25 DPA. These results provided an interesting stage-specific distribution of eQTL in the different cotton subgenomes, suggesting that they exert their effects at specific periods during cotton fiber development.

**Table 2:** Number of significant eQTL (P<0.05) detected per gene across 444 genes.

Number of significant	10DPA	Percent	25 DPA	Percent	All	Percent
(P<0.05) eQTL per gene						
0	15	10.6	35	11.6	50	11.3
1	36	25.4	86	28.5	122	27.5
2	42	29.6	82	27.2	124	27.9
3	28	19.7	51	16.9	79	17.8
4	17	12.0	31	10.3	48	10.8
5	3	2.1	8	2.6	11	2.5
6	1	0.7	7	2.3	8	1.8
7	0	0.0	2	0.7	2	0.5
Total	142		302		444	

Genomic sequence data for tetraploid cotton are presently limited for the determination of whether an eQTL is *cis*- or *trans*-acting on a large scale. However, the positional *cis*-/*trans*-acting eQTL can be estimated by comparing the chromosomal location of each transcript and position of its eQTL. For integration of genetic mapping with differential gene expression profiling, we developed and mapped 23 expressed sequence tag (EST)-derived locus-specific markers based on different EST sequences in *G. hirsutum* and *G. barbadense*. The other seven EST-simple sequence repeat (SSR) markers had been mapped previously [35]. The mapping of these 30 differential genes are presented in Table S3. To identify the eQTL location of the 30 genes, their expression values were extracted from 28 k microarrays for linkage mapping. Out of these 30 functional markers, eight (26.7%) agreed with the positions of eQTL within 18 cM (Table S3), which were considered to be regulated in *cis*, such as the quinate hydroxycinnamoyltransferase (HCT) gene eQTL on chr. A10. The rest most likely were *trans*-eQTL, such as 28k_275_A03_CS (Ca^2+^-ATPase) on chr. A11. These results showed that the majority of eQTL in cotton regulated differential expression in a *trans*-acting manner in this population.

We observed eight genes associated with fiber quality QTL within 20 cM intervals. The genes encoding cell wall structural proteins including kinesin-like protein and alpha-tubulin 10 were mapped in the intervals of QTL *qFL-A1* and *qFS-A5*, respectively; meanwhile, genes for Ca^2+^-ATPase, NADPH∶quinone oxidoreductase (NQR), and non-symbiotic hemoglobin 2 (Hb2) involved in calcium signaling pathway and reactive oxygen species (ROS) metabolism were localized within QTL for fiber fineness, *qFM-D12*, *qFM-D4* and *qFM-D1*. However, these results are presently limited for precise identification of fiber quality genes on a large scale, especially for mapping fiber quality QTL.

### eQTL hotspots

**Figure 3:**
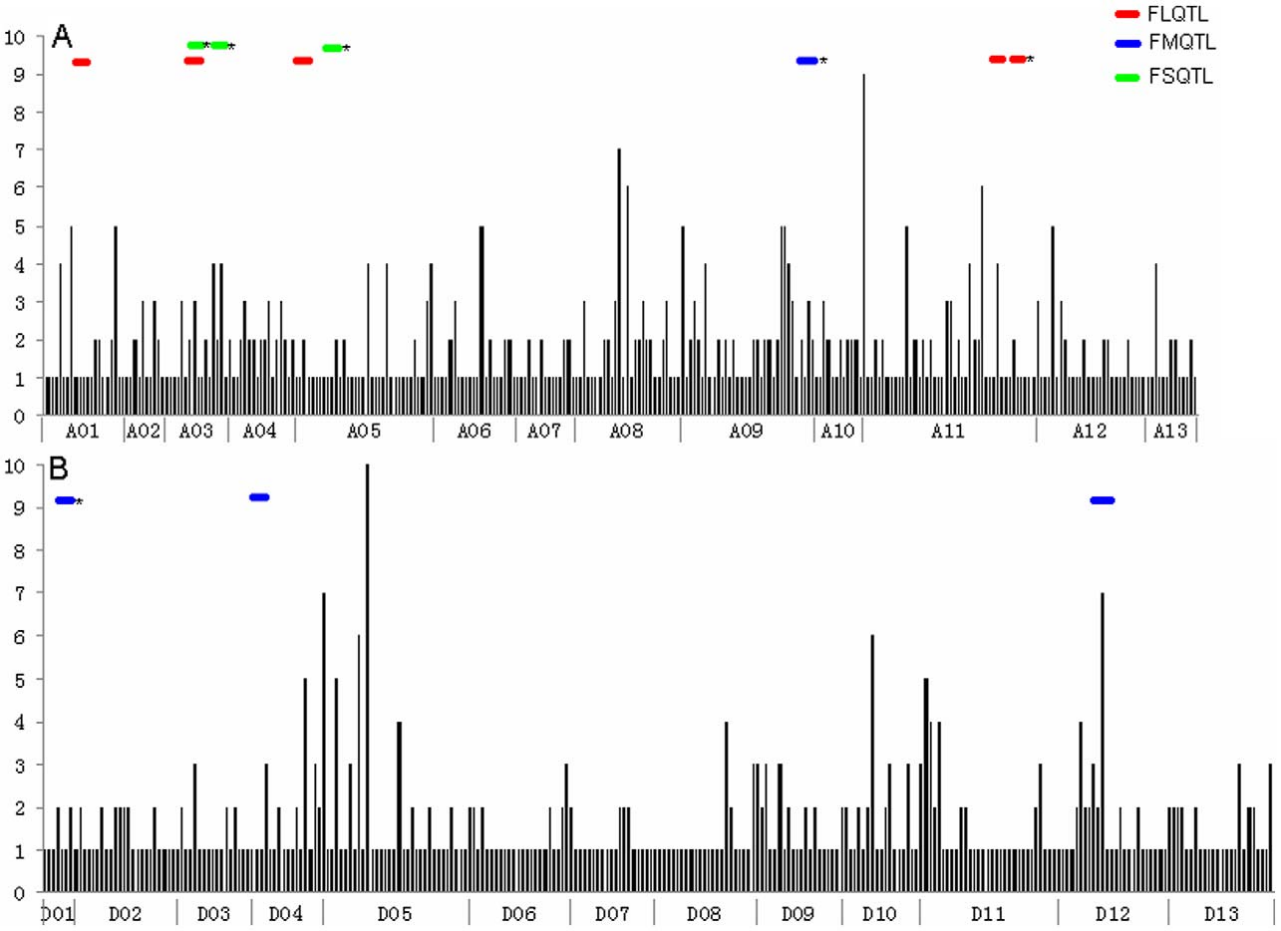
Comparative distributions of eQTL and fiber quality QTL in cotton. The eQTL density for fiber genes across 13 A- (A) and 13 D-subgenome chromosomes (B). The x axis shows eQTL genetic location on chromosomes, and y axis represents the number of eQTL per cM. QTL for fiber length (FL), strength (FS), and micronaire (FM) are indicated in color bars. *indicates main-effect QTL. Detailed comparisons between locations of eQTL and fiber QTL are plotted in the genetic map in Figure S2.

In this study, the eQTL were not evenly distributed across the genome. The eQTL densities for fiber genes and fiber quality QTL across chromosomes are presented in Figure 3. The largest number (80) of eQTL was mapped on chr. A11, but only 9 eQTL were on chr. D1. It is clear that some regions on chr. A1, A3, A6, A8, A9, A11, D5, D11 and D12 had larger numbers of eQTL, as indicated by the thick horizontal lines. Thus, there were specific genomic regions where the eQTL clustered (hotspots), and a nearly 2-fold higher number of hotspots were distributed in the At than in the Dt (Table 3). These eQTL hotspots appeared to be unequally distributed in the cotton subgenomes during specific fiber developmental stages, where the majority of hotspots during the fiber fast elongation were on chr. A5, A10, D11 and D12, while at the SCW synthesis stage, most of the hotspots were on chr. A1, A8, A9, A11 and D5. The results suggested that regulatory control for many cotton fiber genes exist in short chromosomal intervals that are either gene-rich or that recombine infrequently.

**Table 3:** Details of eQTL hotspots detected across all chromosomes.

Chr	Position/	No.	No.	No.	Positive	Negative	Involving	Fiber
	Interval(cM)^a^	Total	10 D	25D	(Gb%)^b^	(Gh%)^c^	TFs^d^	QTL^e^
A1	42.2–45.3	6	1	5	2(33%)	4(67%)		
	54.2–55.8	6	1	5	2(33%)	4(67%)		*qFL-A1*
	84.8	5		5	5(100%)			
D2	43.1–45.4	6	2	4	6(100%)		zf-C3HC4	
A3	69.2–73.1	5	2	3	2(40%)	3(60%)		*qFS-A3-1*
	77.1–78	6	1	5	1(17%)	5(83%)	zf(B-box)	*qFS-A3-2*
A4	86.8–89.3	6		6	5(83%)	1(17%)		
D4	90	5	1	4	1(20%)	4(80%)	bZIP	
A5	108.2–112.9	5	1	4	4(80%)	1(20%)		
	139.6–141.6	5		5	2(40%)	3(60%)		
	152.1–156.1	5	5		2(40%)	3(60%)		
D5	0	7	2	5		7(100%)	ERF	
	40.6–44.6	6	2	4	3(50%)	3(50%)		
	50.3–53.6	10		10	10(100%)		zf-CCCH	
	59.1–60.9	11	1	10	10(91%)	1(9%)		
	77–79.8	5	2	3	4(80%)	1(20%)		
A6	103.1–107.1	8		8	5(63%)	3(38%)	NAC091	
A8	27.4–31.4	5	0	5	5(100%)		RAP2-4	
	37–39.7	14		14	13(93%)	1(7%)		
	63.2–65.2	5	1	4	5(100%)			
	94.8–96.8	5	2	3	3(60%)	2(40%)		
D8	112.8–114.3	6		6	5(83%)	1(17%)	NST1	
A9	0	5		5		5(100%)	Salt tolerance zf	
	6–9.8	6	1	5	1(17%)	5(83%)	Salt tolerance zf	
	15.3–18.9	5		5	1(20%)	4(80%)	AP2; zf(B-box)	
	78.1–81.3	5	1	4	4(80%)	1(20%)		
	96.6–98.6	7		7	7(100%)		HSP	
	101.5–105.5	9		9	8(89%)	1(11%)	NST1;NAC1	
D9	0–2	5	1	4	1(20%)	4(80%)	zf-C3HC4	
A10	42.9–44.9	5	5		2(40%)	3(60%)		
D10	58.6	6	1	5	5(83%)	1(17%)	GRAS	
A11	0–2	10	1	9	10(100%)			
	53.4–55.7	7	1	6		7(100%)	RAP2-4;ERF;bZIP	
	64.1–66.7	5		5		5(100%)	GRAS;bZIP	
	78.1–81.7	5	1	4		5(100%)	GRAS	
	100.5–102.5	6		6	1(17%)	5(83%)		
	104.7–106.7	8	1	7		8(100%)		
	115.5–119.4	7	2	5		7(100%)		*qFL-A11-1*
D11	14.1	5	3	2	3(60%)	2(40%)		
	16.1–20.1	10	10		10(100%)			
A12	23–27	6		6	3(50%)	3(50%)		
D12	35.8–38.8	8	2	6	5(63%)	3(38%)	HSP	
	61.8–62.9	9	7	2		9(100%)		*qFM-D12*
A13	35.3–36.2	5		5	5(100%)		NAC091	
D13	96.1–101.1	5		5		5(100%)		
	111.3–114.9	5	2	3	2(40%)	3(60%)		
At	30	187	27	160	98(52%)	89(48%)		
Dt	16	109	36	73	65(60%)	44(40%)		
Total	46	296	63	233	163(55%)	133(45%)		

a These eQTL hotspots were apparent with greater than 0.5% of the total number (916) of eQTL identified genome-wide localize to a 4-cM window;

b Number of eQTL with a positive additive-effect value (Percentage of eQTL favorable alleles from Hai7124);

c Number of eQTL with a negative additive-effect value (Percentage of eQTL favorable alleles from TM-1);

d Transcription factor within eQTL hotspots;

e QTL for fiber qualities were co-localized with eQTL hotspots within 10 cM.

The abbreviations used are: Zinc finger (zf); Ethylene-responsive transcription factor (ERF); Heat shock protein (HSP); NAC secondary wall thickening promoting factor1 (NST1).

At 46 eQTL hotspots, 55% of the 296 eQTL had a positive additive effect for which the Hai7124 allele toward increased expression, and 45% had a negative additive effect (favorable alleles from TM-1) (Table 3). A strong bias (>90%) towards favorable effects from Hai7124 alleles was detected for hotspots on chr. A1, A8, A9, A13, D2, D5, D11 and in the telomeric region of chr. A11. There was also a strong bias (>90%) towards favorable effects from TM-1 alleles on chr. A11, D5, D12, D13, and in the telomeric region of chr. A9. Interestingly, the number of observed eQTL, whether bias towards Hai7124 or TM-1, in the A-subgenome was greater than those in the D-subgenome (Table 3).

eQTL hotspots can facilitate the identification of underlying biological links. We found that a large proportion of chalcone synthase (CHS) genes that were extensively mapped to the eQTL hotspots regions, involving chr. A5, A11, D10, D11 and D12 during fiber cell elongation, and chr. A1, D8, A9 and D9 during SCW formation. The two largest hotspots related to CHS were on chr. D11 and D12 during fiber elongation, and one large SCW formation hotspot related to CHS was on chr. A1. Some CHS genes were co-localized in the hotspot regions with genes encoding plant hormones, transcription factors and cell wall enzymes, such as responsive to abscisic acid 1B (Rab1B), ACC synthase 6 (ACS6) on A9, NAC secondary wall thickening promoting factor1 (NST1) on D8, Zinc finger (C3HC4-type) family protein on D9, β-1,3-glucanase in the telomeric region of chr. A11, and invertase and polygalacturonase (PG) on chr. D11. These results implied that CHS may regulate fiber cell development through interaction with plant hormones and cell wall enzymes.

A biologically meaningful eQTL hotspot would represent the location of a master transcriptional regulator that controls the expression of a suite of fiber genes acting in the same biological process. We found genes encoding several transcriptional factors in the hotspot regions (Table 3), including AP2/ERF, Zinc finger, GRAS, bZIP, HSP and NAC family transcriptional regulators. Thus, these transcriptional regulators which reside in the fiber quality QTL regions may be crucial in regulating cotton fiber development.

### Combining QTL mapped for fiber qualities and eQTL

Association analysis between phenotypic variation and eQTL would be useful in dissecting the genetic factors controlling fiber quality properties. By comparative analysis of eQTL and stable QTL for fiber qualities in the present study (Figure S2), especially for eQTL hotspots within the regions of QTL for qualities (Table 3), we identified some eQTL genes co-localized with QTL for fiber qualities (Table 4), suggesting that they may play a part in mediating the development of fiber phenotypes. In addition, the genes underlying qualities with no functional annotations were also listed in Table S4. Some genes differentially regulated at 10 and 25 DPA were associated with fiber quality properties; most noteworthy, the fiber gene expression variation at 25 DPA predominantly affected fiber quality properties. Two eQTL hotspots on chr. A3 co-localized with *qFS-A3-1* and *qFS-A3-2* encode proteins primarily involved in oxidation reduction.

**Table 4:** Identifying eQTL genes co-localized with QTL for fiber qualities.

Stages	Co-localized QTL	Array ID	Gene Name	Putative function
10 DPA	*qFL-A1*	28k_093_F01	Transferase family protein	-
	*qFL-A3*	28k_094_F09	XH/XS domain-containing protein	-
	*qFL-A5*	28k_045_H06	Serine-threonine protein kinase	Catalytic activity
	*qFL-A11-1*	28k_172_A10	NADH dehydrogenase subunit 1	Oxidation reduction
		28k_196_G03	CYP71A16	Oxidoreductase activity
	*qFS-A3-2*	28k_142_D03	GASA-like protein	Regulating H_2_O_2_ levels
	*qFM-A9*	28k_110_B01	Probable carbohydrate esterase	Hydrolase activity
		28k_208_A09	Polygalacturonase inhibiting protein 1	Protein binding
	*qFM-D12*	28k_047_B08	CHS	Flavonoid biosynthesis
		28k_073_A01	CHS	Flavonoid biosynthesis
		28k_181_G05	Chromatin remodeling complex subunit	ATP binding
		28k_257_D01	CHS	Flavonoid biosynthesis
25 DPA	*qFL-A1*	28k_097_H05	Arabinogalactan peptide 20	-
		28k_123_C04	PB1 domain-containing protein	-
		28k_144_D11	Speckle-type POZ protein	Protein binding
		28k_152_A03	Putative GTP-binding protein	Response to gibberellin
	*qFL-A1,qFM-D12*	28k_013_A02	CHS	Flavonoid biosynthesis
		28k_079_H10	CHS	Flavonoid biosynthesis
	*qFL-A5,qFL-A11-2*	28k_166_C10	Cellulose synthase-like A2 (CSLA02)	Cellulose synthase activity
	*qFL-A11-1*	28k_087_B10	ABA-responsive protein	ABA signaling pathway
		28k_275_A03	Calcium-transporting ATPase	Calcium ion transport
		28k_290_B01	ACS6	Ethylene biosynthesis
		28k_298_E02	Nudix hydrolase homolog 4 (NUDT4)	-
	*qFL-A11-2*	28k_294_H02	Dehydratase	-
		28k_302_B12	Calmodulin-binding family protein	Calcium ion signaling
		28k_098_C10	NAC domain protein (NAC1)	Regulation of transcription
		28k_245_C05	PP2C family protein	Protein dephosphorylation
	*qFS-A3-1,qFS-A3-2*	28k_056_B04	NADH dehydrogenase subunit 1,5	Oxidation reduction
	*qFS-A3-2*	28k_064_D09	NADH dehydrogenase subunit 5	Oxidation reduction
		28k_071_D02	NADH dehydrogenase subunit 1	Oxidation reduction
		28k_144_D04	LRR-RLK	-
		28k_211_E10	FAD-binding domain-containing protein	Oxidation reduction
		28k_288_G01	ABC1 family protein	Protein phosphorylation
		28k_302_B01	Zinc finger (B-box type) family protein	Zinc ion binding
		28k_303_C11	GDSL-motif lipase	Lipase activity
	*qFM-A9*	28k_102_C09	ADL4	Response to cadmium ion
		28k_121_B04	ATP binding/phenylalanine-tRNA ligase	-
		28k_158_D06	24-sterol C-methyltransferase (SMT2-1)	BR biosynthetic pathway
		28k_260_H12	NAD(P)H dehydrogenase (ND1)	Oxidation reduction
	*qFM-D1*	28k_233_G07	Probable disease resistance protein	ATP binding
	*qFM-D12*	28k_082_G03	Abscisic acid-responsive HVA22 protein	ABA signaling pathway
		28k_209_H05	TCP family transcription factor	Regulation of transcription

These transcripts of differentially expressed genes were co-localizing with phQTLs in the overlapping intervals, reference Figure S2 in detail. Gene Ontology (GO) annotations and searches of the literature were conducted to identify putative function of differentially expressed genes.

It should be noted that the most significant QTL for fiber length and fineness were located on chr. A11 and A9, coinciding with the two highest numbers of eQTL (80 and 70) or hotspots (7 and 6) across all chromosomes. On the same chr. A11 there was a strong bias (>90%) towards positive effects from TM-1 alleles in five eQTL hotspots, whereas, QTL for FL *qFL-A11-1* and *qFL-A11-2* with positive effects from longer staple parent Hai7124 alleles increased FL. Co-localizing genes residing in the two QTL included those encoding ACS6, PP2C family proteins, NAC domain protein (NAC1), calmodulin-binding family proteins and calcium-transporting ATPase. These down-regulated genes in Hai7124 underlying genetic and expression loci may play a part in superior FL. There were two eQTL hotspots neighboring *qFL-A11-1* that encode proteins involved in oxidoreductase activity, including NADH dehydrogenase, Ca^2+^-ATPase, ATP6, fatty acid desaturase 8 (FAD 8) and alcohol dehydrogenase. Additionally, there was a strong bias (89%) towards positive effects from Hai7124 alleles in two eQTL hotspots on chr. A9 neighboring a main-effect QTL *qFM-A9*; and this QTL was associated with fineness due to positive effects of alleles from the Hai7124 parent with finer fiber traits. Thus, up-regulated genes in Hai7124 at *qFM-A9*, such as the genes for dynamin-like protein 4 (ADL4) and NAD(P)H dehydrogenase (ND1), play a positive role in fiber fineness, whereas the down-regulated genes for carbohydrate esterase and polygalacturonase inhibiting protein 1 (PGIP1) may play a negative role in fiber fineness. The eQTL hotspot genes encode proteins, including transcription regulators such as Zinc finger, AP2, NST1 and NAC1, and cell wall enzymes β-1,3-glucanase and glucan endo-1,3-beta-glucosidase-like protein 4 (E13L3). These eQTL hotspots neighboring the QTL for fiber qualities intervals also produced some candidate genes for regulating fiber length or fineness.

Interestingly, some common eQTL genes appeared to associate with different QTL, such as the gene for cellulose synthase-like protein A2 (CSLA02) commonly associated with *qFL-A5* and *qFL-A11-2*. NADH dehydrogenase genes involved in oxidoreductase activity associated with reactive oxygen species (ROS) metabolism were commonly co-localized with QTL for FL, FM and FS. Meanwhile, a suite of genes encoding key CHS involved in flavonoid biosynthesis co-localized with QTL for FL and FM; several protein kinase genes appeared to correlate with FL and FS (Table 4), implying their crucial roles in common molecular events in developmental processes determining these fiber quality traits. In addition, the *GAST1*-like genes regulating H_2_O_2_ levels were mapped in the homoeologous regions of fiber QTL *qFS-A3-2* and *qFL-A11-2*, such as 28k_113_E06_CS and 28k_142_D03_CE.

### QRT-PCR analysis for selected fiber genes

**Figure 4:**
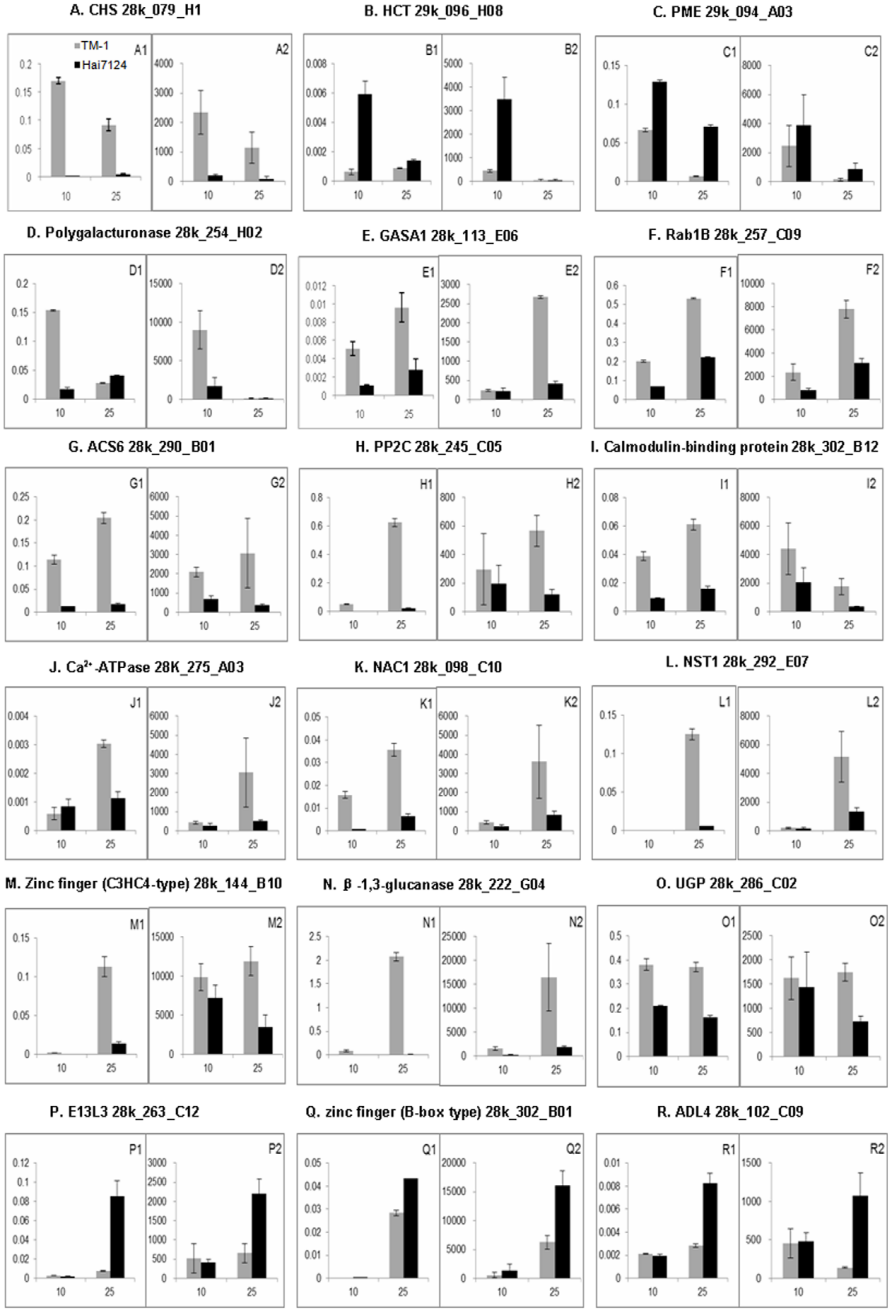
qRT-PCR validation of selected differentially expressed genes. The x axis represents two developmental periods (10 and 25 DPA), and y axis indicates the relative expression value from qRT-PCR (A1-R1) and signal intensity from microarray (A2-R2) in TM-1 and Hai7124 fibers. Data shown are the means of three biological replicates. Error bars indicate ± SD.

Based on eQTL co-location and hotspots, we selected 18 candidate genes to confirm differential expression from our microarray analysis in TM-1 and Hai7124 (Figure 4). For the majority of the tested genes, the qRT-PCR results confirmed the microarray analysis. These genes are primarily involved in flavonoid biosynthesis, plant hormones and calcium signaling pathways, transcription factors, and cell wall polysaccharides biosynthesis or modification. Several genes were preferentially expressed in the fiber fast elongation stage (10 DPA) (Figure 4A–D). The expression of cell wall loosening enzyme gene *PME* was observed to decrease sharply at SCW synthesis in TM-1, but decreased slowly in Hai7124. Interestingly, the expression of most of the tested genes in TM-1 increased rapidly when cell elongation ceased at 25 DPA, whereas in Hai7124 they rose slowly (Figure 4E–N). These genes were up-regulated during 25 DPA in TM-1 but were repressed in Hai7124 that produced longer fibers, suggesting that these genes, such as those encoding Rab1B, ACS6, NAC and Zinc finger (C3HC4-type) family transcription factor, β-1,3-glucanase, play a specific role in preventing fiber elongation or promoting SCW synthesis. The expression of three genes encoding glucan endo-1,3-beta-glucosidase-like protein 4 (E13L3), Zinc finger (B-box type) family protein and dynamin-like protein 4 (ADL4) had a sharp increase in Hai724 at 25 DPA (Figure 4P–R). Thus, confirmation by qRT-PCR analysis allowed us to identify potentially important DEGs regulating fiber cell elongation or SCW synthesis.

## Discussion

**Figure 5:**
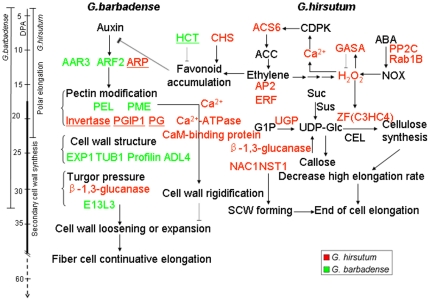
Molecular mechanisms of differential cotton fiber length in *G. hirsutum* and *G. barbadense.* * G. barbadense* showed a longer transition stage with overlapping fiber elongation and secondary cell wall (SCW) synthesis than *G. hirsutum*. Metabolic pathways were differentially regulated during fiber development, and pectin and cellulose metabolism in particular affected fiber elongation and SCW biosynthesis. In the SCW synthesis stage (represented by 25 DPA), the auxin signaling pathway and pectin modification genes were up-regulated in *G. barbadense* compared with *G. hirsutum*, and the SCW synthesis genes were repressed. In *G. hirsutum* pathways producing cellulose were enhanced, while auxin and pectin modification were repressed. Selected genes were also analyzed by qRT-PCR as in Figure 4. Red represents up-regulated in TM-1, green represents up-regulated in Hai7124. Underlined genes were differentially expressed only at 10 DPA. The abbreviations used are: day post-anthesis (DPA); chalcone synthase (CHS); quinate hydroxycinnamoyltransferase (HCT); antiauxin-resistant 3 (AAR3); auxin response factor 2 (ARF2); auxin-responsive protein (ARP); GAST1 protein homolog 1 (GASA1); 1-aminocyclopropane-1-carboxylic acid (ACC); ACC synthase 6 (ACS6); Ca^2+^-dependent protein kinase (CDPK); calmodulin (CaM)-binding protein; ethylene-responsive transcription factor (ERF); protein phosphatase 2C family protein (PP2C); abscisic acid (ABA); responsive to abscisic acid 1B (Rab1B); NADPH oxidase (NOX); Cell wall ezymes: pectin methylesterase (PME); pectate lyase (PEL); polygalacturonase (PG); polygalacturonase inhibiting protein 1 (PGIP1); glucan endo-1,3-beta-glucosidase-like protein 4 (E13L3); Cell wall structure: expansin (EXP1); tubulin beta-1 (TUB1); dynamin-like protein 4 (ADL4); Cellulose synthesis: Glucose 1 Phosphate (G1P); sucrose synthase (Sus) sucrose (Suc); Zinc finger (C3HC4-type) family protein (ZF (C3HC4)); UDP-glucose pyrophosphorylase (UGP); Cellulose synthase (CEL); NAC secondary wall thickening promoting factor1 (NST1).

*Gossypium barbadense* produces superior fibers than *G. hirsutum*, yet the regulatory mechanisms governing its difference in fiber qualities are not well known. In this study, the dynamic analysis of fiber growth and gene expression variation among different time points between *G. barbadense* and *G. hirsutum* suggested two respective periods (10 and 25 DPA) that were crucial for further identification of fiber quality genes. Furthermore, we integrated large-scale microarray-based gene expression profiling with linkage analysis in cotton and found 916 eQTL in each stage (10 and 25 DPA) after assessing genome-wide significance. These eQTL represent a large source of functional genes for the fiber QTL that were mapped from the combined four-year data in this study. The fiber length measurements suggested that significant differences between *G. barbadense* and *G. hirsutum* occurred at the overlapping period of elongation and SCW formation, which was longer in *G. barbadense* (19–33 DPA) than that in *G. hirsutum* (19–23 DPA) (Figure 5). This result agrees with previously reported results, and the duration of transition stage may determine cotton fiber quality properties [2]–[4], [6], [9], [17]. At this transition stage, *G. hirsutum* is known to have a higher rate of cellulose deposition than *G. barbadense* [3]–[4], although *G. barbadense* maintains a higher rate of fiber elongation than *G. hirsutum*. Interestingly, a large proportion of differentially regulated genes at 25 DPA in this study appeared to be associated with fiber length, strength and fineness (Table 4), which enabled us to further elucidate the molecular mechanisms controlling differential fiber length in *G. hirsutum* and *G. barbadense* (Figure 5).

### Molecular basis of prolonged fiber elongation in *G. barbadense*

Plant hormones and cell wall enzymes play a prominent role in mediating fiber cell elongation. Two sets of microarry platforms showed similar results with significantly increasing trends of up-regulated genes in *G. barbadense* from 10 to 25 DPA (82 vs. 192 and 29 vs. 113, respectively) when compared with *G. hirsutum.* The expressions of fiber cell elongation related genes decreased slowly in *G. barbadense*, suggesting fiber elongation related genes had long-term expression and supported a longer duration of elongation in this cotton species at the transition stage. Of these, auxin signaling pathway and pectin modification genes antiauxin-resistant 3 (*AAR3*) and pectin methylesterase (*PME*) preferentially expressed at the fast elongation stage were up-regulated in *G. barbadense* compared with *G. hirsutum* at 25 DPA and showed similar time-course expression patterns (Figure S1). Previous reports have indicated that auxin promotes fiber initiation and early stages of elongation [7], [11], [13], [42], but little is known about their roles at the transition stage. The cell-wall loosening enzyme PME was repressed in the gain-of-function Aux/IAA mutant (*axr3-1/iaa17-1*) for auxin resistant 3 in *Arabidopsis* [43]. Recent studies suggest that genes regulating pectin play key roles in controlling fiber cell elongation, comparative proteomics confirmed that biosynthesis of pectic precursors was important for cotton fiber and *Arabidopsis* root hair elongation [16]; pectin synthesis genes were the most significantly differentially expressed categories during fiber elongation, and the expression levels of pectin modification genes showed high correlations with specific fiber properties [18]; transgenic modifications also confirmed that *GhPEL* gene encoding pectate lyase positively regulated fiber length [44]. These data indicate that auxin may regulate fiber cell elongation through pectin modification (Figure 5).

Turgor-driven cell expansion is required for cotton fiber elongation at the transition stage. The two novel genes encoding glucan endo-1,3-beta-glucosidase-like protein 4 (*E13L3*) involving callose binding activity, which were highly expressed in *G. barbadense* fibers at 25 DPA, led to an increase in callose accumulation to regulate plasmodesmata closure [9], [45]. By contrast, we found several genes for β-1,3-glucanase, which degrade callose to regulate plasmodesmata opening, that had low levels of expression in *G. barbadense* fibers [9]. These two gene families control duration of plasmodesmata closure to sustain high turgor for a longer period, consistent with longer fiber elongation stage in *G. barbadense* than in *G. hirsutum*. Therefore, these results suggest auxin signaling pathway and cell wall enzymes provide a basis for *G. barbadense* fiber to sustain elongation.

### Molecular basis for the early termination of fiber elongation in *G. hirsutum*

Ethylene and abscisic acid (ABA) may regulate cotton fiber cell development through increasing H_2_O_2_ levels. Comparative analysis of eQTL and QTL for fiber qualities identified that ethylene, ABA and calcium signaling pathway genes mapped in eQTL hotspots co-localized with the major QTL for FL, such as genes encoding ACS6, PP2C and ABA-responsive proteins, CaM-binding protein and Ca^2+^-ATPase. A large proportion of ethylene and ABA signaling pathway genes sharply increased from 10 to 25 DPA and were significantly up-regulated in *G. hirsutum* fibers at 25 DPA when compared with *G. barbadense* (Figure 4). These over-expressed genes in *G. hirsutum* fibers are associated with H_2_O_2_ regulation. Previous studies indicated that both of ethylene and ABA can promote H_2_O_2_ production in cotton fiber and *Arabidopsis* [46]–[47], and ABA is considered a signal of secondary wall thickening during cotton fiber elongation [48]. The present study also showed a *GAST1-like* gene (28k_113_E06) was preferentially expressed in *G. hirsutum* compared with *G. barbadense* in the SCW stage. *GAST1-like* (GASA1) is a member of the gibberellin-induced, cysteine-rich protein family previously shown to be induced by H_2_O_2_ [49]. These results imply a higher level of H_2_O_2_ in *G. hirsutum* fiber cells than *G. barbadense* at 25 DPA, functioning as a developmental signal in the onset of SCW synthesis and the end of cell elongation in cotton fibers [15], [50]. Thus, ethylene and ABA may trigger the onset of SCW synthesis and the end of cell elongation in cotton fibers through increasing H_2_O_2_ levels.

A previous study showed that ethylene promotes cotton fiber cell elongation [14], which was based on experiments using low concentrations of ethylene in the ovule culture at the early stage of fiber elongation. Other studies have shown that low concentrations of ethylene can modulate low-level H_2_O_2_ production that is important for cell elongation in *Arabidopsis* and cotton [15], [46], [51]. Our study also showed that two *GAST1-like* genes (28k_142_D03 and 28k_153_G08) which were preferentially expressed at 10 DPA in *G. hirsutum*, may promote fiber elongation through modulating H_2_O_2_ levels. 28k_142_D03 was mapped within an eQTL hotspot associated with a major QTL for FS on A3, and 28k_153_G08 was mapped within 2 cM with a gene encoding glutamate decarboxylase 4 (GAD4) involved in the calcium signaling pathway. H_2_O_2_ is generated by ABA-induced activation of NADPH oxidase and mediates activation of the Ca^2+^ channel pathway in *Arabidopsis* [47], [51]. In this study, several Ca^2+^ signaling pathway genes were up-regulated in *G. hirsutum* than *G. barbadense* at 25 DPA, implying high levels of Ca^2+^ in *G. hirsutum* fiber cells, which can increase the cell wall rigidity by binding de-esterified pectin and restrict further wall expansion [52]. The metabolic pathways at the transition stage in *G. hirsutum* streamed into high-rate cellulose synthesis, and fiber elongation trended towards cessation. Our results showed that the expressions of Zinc finger (C3HC4-type) and NAC family transcription factors sharply increased during *G. hirsutum* fiber SCW synthesis (Figure 4K–M). Zinc finger (C3HC4-type) family transcription factors also regulate cellulose synthesis via oxidation of the zinc-binding domains [53]. NST1 is a key regulator of the formation of secondary walls in *Arabidopsis* [54]. This cascade of transcription factors regulates expression of genes to promote the biosynthesis of secondary walls. These results support that ABA and ethylene may inhibit fiber elongation and enhance SCW synthesis through regulating high levels of H_2_O_2_ and calcium at the transition stage, which is a possible basis for the termination of fiber elongation.

Most of the genes in favonoid biosynthesis pathway were up-regulated in TM-1 during fiber elongation. Of these, a large percentage of CHS genes were extensively mapped in the regions of eQTL hotspots on chr. A1, A5 and A11 associated with significant QTL for fiber length. Previous studies demonstrated that flavonoids accumulation act as negative regulators of auxin transport when the gene encoding quinate HCT in lignin synthesis is repressed [55]–[56]. Since ethylene also modulates flavonoid accumulation [57], CHS and ethylene therefore can affect auxin transport by enhancing flavonoid accumulation, which may negatively regulate fiber cell elongation. These results support down-regulated expression of these fiber elongation related negative regulators in *G. barbadense* during the transition stage likely help fiber cells to maintain auxin levels, which is another basis for *G. barbadense* fiber cell to sustain elongation.

In conclusion, the high-level and long-term expression of auxin regulators, pectin modification and turgor related genes may promote continuing fiber cell elongation by cell wall loosening and enlargement at the transition stage; and down-regulation of the ABA and ethylene signaling pathway and SCW biosynthetic gene expression in *G. barbadense* helps the fiber cells to maintain the high elongation rate for sustained elongation (Figure 5). These may be the two major causes for fiber cell sustained elongation at the transition stage. These molecular mechanisms may facilitate the longer duration of the transition stage in *G. barbadense* and ultimately determine superior fiber quality properties such as fiber length, strength and fineness.

To our knowledge this study is the first to integrate developmental genetics and genomics of a single cell type in plants, and which provides an opportunity to better understand how complex plant hormones-signaling pathways are integrated to control cotton fiber qualities. Further functional validation of the currently large percentage of genes with unknown functions in fiber QTL will provide novel clues for a fiber cell model involving elongation or cellulose biosynthesis. With the completion of cotton genomic sequencing projects in the near future and further localization of genes, many more *cis*-acting eQTL genes residing in fiber QTL should be obtained from the data gained in this study, which will provide a rich resource for studying genes underlying fiber qualities. Genetic improvement of commercial cotton cultivar fiber quality properties is expected to be achieved using these novel gene resources or loci in this study by genetic transformation or marker assisted selection breeding programs.

## Materials and Methods

### Plant materials

TM-1 is a genetic standard accession of *G. hirsutum* developed in the USA, and Hai7124 is a commercial *G. barbadense* Verticillium-resistant cultivar with superior fiber length, fineness and strength. All BC_1_ (TM-1/Hai7124//TM-1) plants were grown in the field during the summer seasons and transferred to the greenhouse during the winter seasons at Jiangpu Cotton Research Station of Nanjing Agricultural University (JCRSNAU), Nanjing, China. All BC_1_ plants were self-pollinated to produce replicate individuals from the BC_1_S_1_ mapping population, which enabled us to perform four independent field trials using the same genotypes. The two parents (TM-1 and Hai7124) and 138 BC_1_S_1_ lines were grown during the summer seasons at JCRSNAU, Nanjing, China in 2003, 2004, 2005 and 2008. Fiber samples of four years were harvested from at least 15 plants of the BC_1_S_1_ lines, and the following fiber quality traits were evaluated by using the HVI Spectrum (Zellweger Uster Corp., Switzerland): 2.5% fiber length (FL, mm), fiber strength (FS, cN/tex) and micronaire reading (FM).

Fiber samples of TM-1 and Hai7124 at 5, 10, 15, 20 and 25 DPA in 2006 were used for 12 k microarray hybridizations, and that from TM-1, Hai7124 and 66 randomly selected out of 138 BC_1_S_1_ lines at 10 and 25 DPA in 2008 for 28 k microarray hybridizations. These samples were collected individually from the 15 plants in the morning (10:00–11:30 AM), and the fiber cells with the exception of those at 5 DPA were carefully dissected from the ovules excised from each boll. All harvested fiber materials were immediately frozen in liquid nitrogen and stored at −70°C.

### Fiber length measurements

To reveal the dynamics of fiber development, seeds of TM-1 and Hai7124 at 5, 10, 15, 20, 23, 28, 33 and 38 DPA were chosen to determine the FL using a previously reported method [58]. Six sample replicate data were processed using Microsoft Excel.

### RNA isolation, amplification and labeling

Three equal-volume pools of each sample of TM-1 and Hai7124 were used for extraction of total RNA. Total RNA was extracted from 5 DPA ovules and 10 to 25 DPA fibers of TM-1 and Hai7124 using a modified hot-borate method [59]. Total RNA was extracted from the 10 and 25 DPA fibers of TM-1, Hai7124 and the 66 BC_1_S_1_ lines using a CTAB method [60]. The RNA was concentrated in isopropyl alcohol, followed by purification using the NucleoSpin® RNA Clean-up Kit from MACHEREY-NAGEL (MN, Germany) and finally checked for quantity and purity. Total RNA was reverse transcribed into double-stranded cDNA using the CbcScript enzyme. The cRNA was then synthesized from the second-strand cDNAs with the T7 Enzyme Mix, and the amplified cRNA products were purified using the RNA Clean-up Kit. The cDNA was synthesized with random primers from cRNA using the CbcScript II enzyme. The cDNA products were purified using a PCR NucleoSpin Extract II Kit (MN, Germany) and labeled with fluorescent dye (Cy5 and Cy3-dCTP) using the Klenow Fragment Polymerase from the cRNA Amplification and Labeling Kit.

### Cotton fiber cDNA microarray hybridization

The 11,692 probes of the 12 k cotton fiber cDNA microarray (GPL2610) and 29,184 probes of the 28 k cotton cDNA microarray platform (GPL8569) were from 5–10 DPA and −3∼25 DPA Upland cotton (*G. hirsutum* L. cv. Xuzhou 142) ovules and fibers, respectively. The Cy3 and Cy5-labeled cDNA were resolved in 80 µL hybridization solution (3×SSC, 0.2% SDS, 5× Denhart's, 25% formamide) and denatured at 95°C for 3 min prior to loading onto the microarray. The slides were hybridized at 42°C overnight and transferred to fresh containers with Wash Solution 1 (0.2% SDS, 2× SSC) at 42°C for 5 min and then immersed in Wash Solution 2 (0.2× SSC) at room temperature for 5 min. All microarrays were scanned with the LuxScan™ scanner, and the array image signals were translated into numeric signals with the LuxScan™ 3.0 software (both from CapitalBio Corp., China). All 12 k cotton cDNA microarray data from this work have been deposited to a MIAME compliant database at the National Center for Biotechnology Information (NCBI) GEO database (GSE18028, GSM450751 to GSM450764).

### Statistical analysis

Statistical analyses of differential gene expression measured in the cDNA microarrays were carried out with open source R software packages available as part of the Bioconductor project (http://www.bioconductor.org). Considering that global gene expression changes may exist across different developmental stages, we applied a linear normalization method to normalize individual channel data. The normalized signal intensity values in the 15 microarray slides from the 12 k platform hybridized with RNA prepared from three biological replicate samples for each developmental stage were further analyzed using the empirical Bayes method in the LIMMA package [41], and they were filtered according to the following criteria: (i) exclude genes with low-intensity signal levels (raw signal value <400); (ii) assignment present in 3 repeats from expression ratios; and (iii) log-transformation results in a fold change greater than 1 with Bonferroni adjusted *P*-value<0.05.

A total of 72 slides including six of the two parents and 66 of the BC_1_S_1_ population at 10 and 25 DPA were obtained from the 28 k microarray platform. The raw signals from the microarrays were normalized linearly based on 72 slides after applying background subtraction. Analysis of DEGs was performed using the empirical Bayes method in the LIMMA package [41], and they were filtered according to the following criteria: (i) assignment present in 3 repeats from normalized signal; (ii) exclude genes with low-intensity signal levels (raw signal value <250); and (iii) log-transformation results in a fold change greater than 1 with expected *P*-value of less than 0.05 by LIMMA [41]. Normalized data from the 66 BC_1_S_1_ samples at each fiber developmental stage were used for further linkage analysis. Gene Ontology (GO) annotation was used to infer biochemical pathways involved in genes by Blast2GO (http://www.blast2go.de).

### Linkage and data analysis

A complete linkage map for all 26 chromosomes was constructed using major microsatellite markers [35]. We selected 654 markers from a complete linkage map, including 2,147 markers with 5-cM intervals between markers on 26 chromosomes. The CIM analysis was implemented using Windows QTL Cartographer 2.5 (http://statgen.ncsu.edu/qtlcart/WQTLCart.htm). LOD scores were calculated at 2-cM intervals throughput the genome for each gene expression. QTL Cartographer was used to establish genome-wide significance using the permutation test, and at least 1,000 permutations were used to establish empirical *P-*values (0.05). QTL for FL, FS, and FM at each year were calculated using the CIM approach [37]. QTL and QTL-by-environment interactions for FL, FS and FM with combined four-year data were evaluated using multi-QTL joint analysis under the framework of penalized maximum likelihood [36].

### QRT-PCR analysis

For qRT-PCR validation, additional biological samples were collected from TM-1 and Hai7124 at 10 and 25 DPA. qRT-PCR analyses were performed on three biological replicates from three independent RNA samples. First-strand cDNA was synthesized from 1 µg total RNA using the M-MLV reverse transcriptase (Promega, USA) according to the manufacturer's instructions. Gene-specific primers were designed according to EST sequences using Primer Premier 5.0 (Table S5) and synthesized by GenScript Corp. (Nanjing, China). The transcript encoding cotton elongation factor (*EF1α*) was amplified as a standard control for qRT-PCR analysis. The DEGs in cotton fiber that had been obtained from the microarray analysis were confirmed by qRT-PCR using iQ SYBR Green Supermix (Bio-Rad, USA) in the iQ5 Multicolor Real-Time PCR Detection System (Bio-Rad, USA). The qRT-PCR cycles were as follows: initiation with a 10-min denaturation at 95°C, PCR amplification for 40 cycles of 10 s at 95°C, 20 s at ∼56°C and 30 s at 72°C. The relative gene expression level was calculated with the equation Y = 2^(−ΔCt)^ (ΔCt = Ct target gene-Ct *EF1α*).

### Molecular mapping

The 23 locus-specific PCR primers were designed according to the EST sequences in microarray (Table S6). The other seven genes including the redundant sequences of SSR-ESTs had been mapped previously in our lab [35]. PCR amplifications were performed as follows: at 95°C for 10 min, 30 cycles at 95°C for 1 min, 55°C for 1 min and 72°C for 90 s, followed by a final extension at 72°C. The polymorphic loci were integrated to our backbone linkage maps using JoinMap 3.0 [61].

## Supporting Information

Figure S1 Temporal expression variation in *G. hirsutum* and *G. barbadense* fibers.
(DOC)

Figure S2 Chromosomal co-localizations of eQTL and QTL for fiber qualities.
(DOC)

Table S1 List of all differentially expressed genes at 10 and 25 DPA.
(XLS)

Table S2 Details of 916 significant eQTLs in cotton genome.
(XLS)

Table S3 Comparison of fiber quality and eQTL mapping with chromosomal locations mapping for 30 probe sets.
(DOC)

Table S4 Identifying candidate genes associated to fiber quality properties.
(DOC)

Table S5 Primers used in this study for qRT-PCR analysis.
(DOC)

Table S6 Gene specific primers used in this study for mapping.
(DOC)
