# Supplementary material for: Molecular Mechanisms of Fiber Differential Development between G. barbadense and G. hirsutum Revealed by Genetical Genomics
Source: PLoS One. 2012 Jan 11;7(1):e30056. doi: 10.1371/journal.pone.0030056 (PMC3256209; doi:10.1371/journal.pone.0030056)
Supplement: Figure S1 — Temporal expression variation in G. hirsutum and G. barbadense fibers. (DOC) [file pone.0030056.s001.doc]

*G. hirsutum*

*G. barbadense*

**Figure S1.** Temporal expression variation in *G. hirsutum* and *G. barbadense* fibers.

TM-1 and Hai7124 fiber was investigated in the curve graphs using log transformed signal (y axis) at five developmental stages (x axis) from 12k cotton fiber microarray, and each point on the graph represents the mean of three biological replications. The abbreviations used are: sterol 24-C-methyltransferase (SMT1); expansin (EXP1); tubulin beta-1 (TUB1); auxin binding protein (GLP1); fiber protein E6 (Fiber E6); fiber protein Fb10 (Fb10); pectin methylesterase (PME); antiauxin-resistant 3 (AAR3); auxin response factor 2 (ARF2).
