# Supplementary material for: Molecular Mechanisms of Fiber Differential Development between G. barbadense and G. hirsutum Revealed by Genetical Genomics
Source: PLoS One. 2012 Jan 11;7(1):e30056. doi: 10.1371/journal.pone.0030056 (PMC3256209; doi:10.1371/journal.pone.0030056)
Supplement: Figure S2 — Chromosomal co-localizations of eQTL and QTL for fiber qualities. (DOC) [file pone.0030056.s002.doc]

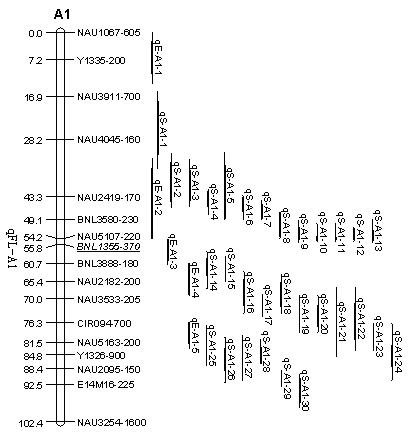

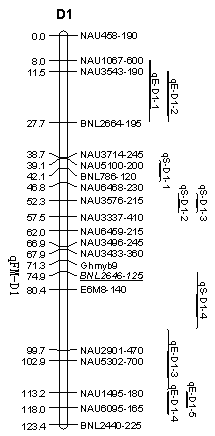

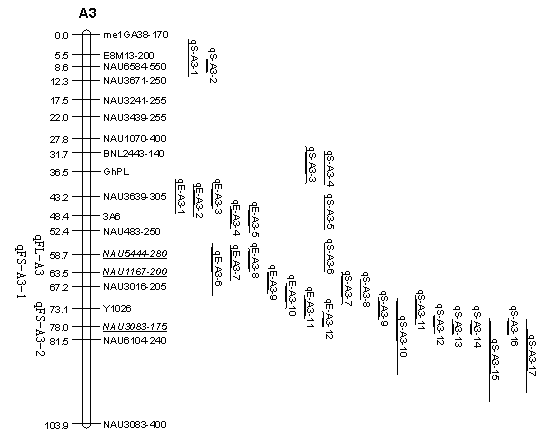

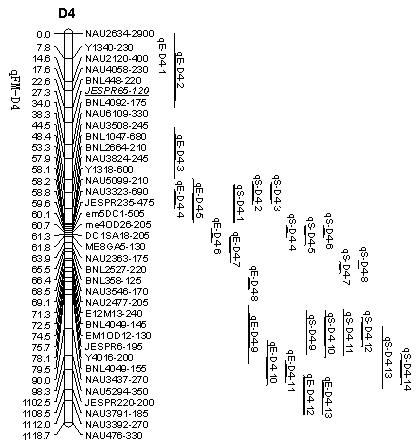


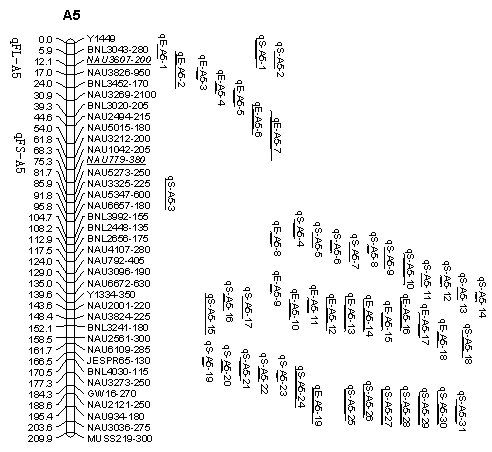

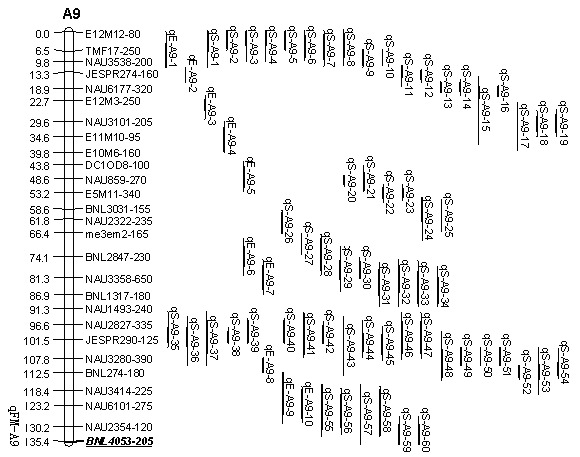


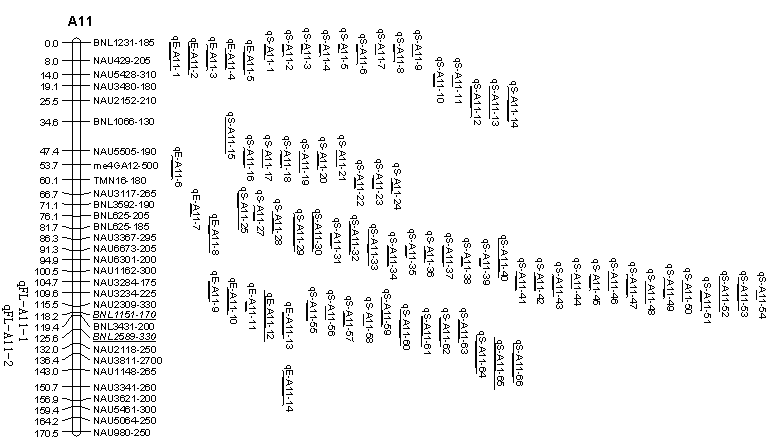


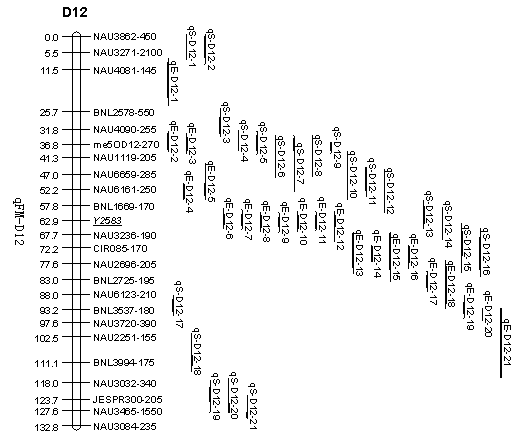


## Figure S2. Chromosomal co-localizations of eQTL and QTL for fiber qualities.

The italicized and underlined markers represent the location of QTL for fiber qualities using multi-QTL joint analysis in the cotton backcross lines, and more detailed information is shown in Table 4. The bars qE-A1-1 and qS-A1-1 on the right of each chromosome represent the location of significant eQTL (*P* < 0.05) at 10 and 25 DPA, respectively. The eQTL information is described in Supplementary Table S2 online. Thick and thin lines indicate the confidence intervals of the QTL corresponding to 1 and 2 LOD units below the maximum LOD score, respectively.
