# Supplementary material for: Molecular Mechanisms of Fiber Differential Development between G. barbadense and G. hirsutum Revealed by Genetical Genomics
Source: PLoS One. 2012 Jan 11;7(1):e30056. doi: 10.1371/journal.pone.0030056 (PMC3256209; doi:10.1371/journal.pone.0030056)
Supplement: Table S3 — Comparison of fiber quality and eQTL mapping with chromosomal locations mapping for 30 probe sets. (DOC) [file pone.0030056.s005.doc]

**Table S3** Comparison of fiber quality and eQTL mapping with chromosomal locations mapping for 30 probe sets

| **Gene Information** | | | **Gene location** | | | **Fiber QTL** | **Cis-eQTL location** | | | | |
| --- | --- | --- | --- | --- | --- | --- | --- | --- | --- | --- | --- |
| **28k_Array ID** | **12k_Array ID** | **Gene name** | **Markers** | **Chr.** | **Pos. (cM)** |  | **Name (18cM)** | **Chr.** | **Pos.** | **LOD** | **R^2^(%)** |
| 28k_273_G04 | CM116G04 | Aminotransferase class IV family protein | Y3876 | A11 | 84.01 |  |  |  |  |  |  |
| 28k_211_E10 | CM047E10 | FAD-binding domain-containing protein | Y3735 | A12 | 69.53 |  |  |  |  |  |  |
| 28k_170_B07 | CM006B07 | Metal ion binding protein | Y3861 | A12 | 137.9 |  |  |  |  |  |  |
| 28k_212_H04 | CM048H04 | Auxin-responsive family protein | Y4292 | A12 | 46.16 |  |  |  |  |  |  |
| 28k_189_C03 | CM025C03 | No Hits | Y3685 | A13 | 59.66 |  |  |  |  |  |  |
| 28k_200_A04 | CM033A03 | alpha-expansin 1 | Y4313 | A13 | 62.31 |  |  |  |  |  |  |
| 28k_165_H09 | CM001H09 | Heat shock protein (HSP) | Y4308 | A5 | 23.13 | *qFL-A5* | 29k_165_H09_CE | A5 | 5.91 | 2.61 | 11 |
| 28k_209_B08 | CM045B08 | Kinesin like protein | Y3734 | A1/D1 | 39.38/59.1 | *qFL-A1* |  |  |  |  |  |
| 28k_096_H08 | CM048C05 | HCT | Y4299 | D10 | 50.38 |  | 28k_096_H08_CE* | A10 | 53 | 8.3 | 35.1 |
| 28k_179_E05 | CM010G08 | CPK28 | Y3486 | D11 | 100.22 |  |  |  |  |  |  |
| 28k_176_F01 | CM009E10 | LRR containing protein | Y3691 | D12 | 49.03 |  |  |  |  |  |  |
| 28k_275_A03 | CM115A09 | Ca2+-ATPase | Y3697 | D12 | 63.72 | *qFM-D12** |  |  |  |  |  |
| 28k_273_C12 | CM116C12 | Germin-like protein 1 | Y3466 | D2 | 58.59 |  |  |  |  |  |  |
| 28k_214_F11 | CM050F11 | MLP-like protein 28(MLP28) | Y3877 | D2 | 56.26 |  |  |  |  |  |  |
| 28k_249_G10 | CM092G10 | Calcium-binding EF hand family protein | Y3711 | D3 | 63.74 |  | 29k_249_G10_CE* | D3 | 64.1 | 3.91 | 14.5 |
| 28k_187_A03 | CM023A03 | Nodulin family protein (NLP) | Y3596 | D5 | 98.91 |  |  |  |  |  |  |
| 28k_257_D05 | CM100D05 | Osmotin-like protein | Y3878 | D5 | 105.88 |  | 29k_257_D05_CE* | A5 | 115 | 3.8 | 15.6 |
| 28k_269_D05 | CM111C03 | Methyltransferase | NAU2170 | A12/D12 | 101.53/79.08 | |  |  |  |  |  |
| 28k_279_E04 | CM122F07 | Glutamine synthetase (GS) | NAU2363 | A4/A4/D4 | 30.90/65.96/63.92 | |  |  |  |  |  |
| 28k_175_F10 | CM011F10 | E3 ubiquitin ligase BIG BROTHER | NAU2816 | D5 | 30.11 |  | 29k_175_F10_CE* | D5 | 22.8 | 3.31 | 11.4 |
| 28k_244_E06 | CM087E06 | CNGC4 | NAU3057 | D1 | 48.08 |  |  |  |  |  |  |
| 28k_193_F11 | CM029F11 | Putative serine/threonine protein kinase | NAU3499 | A8/D8 | 65.10/78.04 |  |  |  |  |  |  |
| 28k_235_C06 | CM071C06 | NAC domain protein NAC5 | NAU3754 | D11 | 66.31 |  |  |  |  |  |  |
| 28k_246_H03 | CM089H03 | No Hits | NAU5418 | A11/D11 | 100.95/102.92 | *qFL-A11-2* |  |  |  |  |  |
| 28k_193_H08 | CM029H08 | alpha-tubulin 10 | Y3719 | A5 | 85.11 | *qFS-A5** |  |  |  |  |  |
| 28k_179_F12 | CM012A01 | Non-symbiotic hemoglobin 2 (Hb2) | Y3568 | D1 | 74.43 | *qFM-D1** | 29k_179_F12_CS | D1 | 57.5 | 5 | 19.6 |
| 28k_267_H07 | CM110H07 | Multicopper oxidase | Y3696 | D11 | 152.29 |  |  |  |  |  |  |
| 28k_211_C10 | CM047C10 | Hypothetical protein | Y4290 | D2/D5 | 53.32/57.82 |  |  |  |  |  |  |
| 28k_263_F02 | CM104H12 | NADPH:quinone oxidoreductase (NQR) | Y4317 | D4 | 7.76 | *qFM-D4* | 29k_263_F02_CE* | D4 | 7.81 | 9.2 | 34.3 |
| 28k_175_C09 | CM011C09 | Elicitor-activated gene 3 (ELI3-1) | Y3593 | D5 | 51.23 |  | 28k_175_C09_CE | D5 | 40.6 | 3.89 | 12.1 |

*within the intervals of 10 cM; 28k_096_H08_CE and 28k_257_D05_CE were detected in the homoeologous regions of A10/D10 and A5/D5.

CE and CS represent eQTL at 10 and 25 DPA, respectively.

The abbreviations used are: Elicitor-activated gene 3 (ELI3-1); Quinate hydroxycinnamoyltransferase (HCT); Cyclic nucleotide-gated ion channel 4 (CNGC4) Calcium-dependent protein kinase 28(CPK28).
