# Supplementary material for: Molecular Mechanisms of Fiber Differential Development between G. barbadense and G. hirsutum Revealed by Genetical Genomics
Source: PLoS One. 2012 Jan 11;7(1):e30056. doi: 10.1371/journal.pone.0030056 (PMC3256209; doi:10.1371/journal.pone.0030056)
Supplement: Table S4 — Identifying candidate genes associated to fiber quality properties. (DOC) [file pone.0030056.s006.doc]

**Table S4.** Identifying candidate genes affecting fiber quality properties

| **Stages** | **Co-localized QTL** | **Array ID** | **Gene Name** | **Putative function** |
| --- | --- | --- | --- | --- |
| 10 DPA | *qFL-A1* | 28k_129_D11 | Unknown protein | - |
|  | *qFL-A11-1* | 28k_072_B10 | No Hits | - |
|  | *qFL-A3* | 28k_163_A11 | No Hits | - |
|  | *qFL-A3,qFS-A3-1* | 28k_158_B09 | Aflatoxin biosynthesis regulatory protein | - |
|  |  | 28k_050_G07 | Hypothetical protein | - |
|  | *qFL-A5* | 28k_196_E03 | No Hits | - |
|  | *qFS-A3-1,qFS-A3-2* | 28k_295_E07 | No Hits | - |
| 25 DPA | *qFL-A1* | 28k_100_D05 | Unknown protein | - |
|  |  | 28k_092_G02 | Unknown protein | - |
|  | *qFL-A11-1* | 28k_211_C10 | Hypothetical protein | - |
|  |  | 28k_061_B08 | No Hits | - |
|  | *qFL-A11-2* | 28k_159_B03 | No Hits | - |
|  | *qFL-A1,qFM-D12* | 28k_067_F09 | Unknown protein | - |
|  | *qFL-A5* | 28k_085_H01 | No Hits | - |
|  | *qFM-A9* | 28k_294_D05 | No Hits | - |
|  |  | 28k_221_C07 | No Hits | - |
|  | *qFS-A3-2* | 28k_118_F02 | No Hits | - |
