# Supplementary material for: Molecular Mechanisms of Fiber Differential Development between G. barbadense and G. hirsutum Revealed by Genetical Genomics
Source: PLoS One. 2012 Jan 11;7(1):e30056. doi: 10.1371/journal.pone.0030056 (PMC3256209; doi:10.1371/journal.pone.0030056)
Supplement: Table S5 — Primers used in this study for qRT-PCR analysis. (DOC) [file pone.0030056.s007.doc]

**Table S5.** Primers used in this study for qRT-PCR analysis

| **Array ID** | **Forward Primer 5'- 3'** | **Reverse Primer 5'- 3'** |
| --- | --- | --- |
| 28k_079_H10 | GGTAAAGAAGCCGCCACAAAG | CCAAAAGAGGGAGTTCCAATC |
| 28k_094_A03 | GCCCGCTCTATCAGGACTAA | TCGCAAGCCTGATGGTTCT |
| 28k_096_H08 | ACACTCCCAAACGAAGTCTATG | CTCGGCAAAAGCACCAAT |
| 28k_098_C10 | TTGATTACCAAATGGAGCAGC | CAAAATGAAGAAAATGGACGG |
| 28k_102_C09 | GAGGCAACAATGTGTCAAGAGG | AACAGTAACCAAGCATAGAGCA |
| 28k_113_E06 | GCCAGCAAGAACAAGGGAA | GAGAAAGATTCGTTCCGATTGTC |
| 28k_144_B10 | ACTTCGTGACGGCAGATAGG | GTGCCGTTTGTAAAGAGCCT |
| 28k_222_G04 | TCCGAGAAACGAGGACATACA | CGTCGTGCCTAACCCAAAT |
| 28k_245_C05 | AGGGCATAATCCAGGTGTC | GTCTACGGCGGTTTCATCG |
| 28k_254_H02 | TAAGCACAAGGCATCCCACC | AGTGGCATCAAAGTGACAGAAAT |
| 28k_257_C09 | CTTATTGGAGATTCAGGTGTTGG | TTCTTGTCCAGCAGTGTCCC |
| 28k_263_C12 | CTCGTCCGTGAACCCAATC | AACCCCACAATAACATCAATCC |
| 28k_275_A03 | TTATTCAAGGTACGGCTGCTA | TCAAACAAATCCTGTGGCTC |
| 28k_286_C02 | ATCCATCAACTAAGGTGTAAAGGTC | TGTGGGTCAACCTGAATGCT |
| 28k_290_B01 | CGGCTCTGTTCCAAGACTATCAT | TGTTCTCCACCTCAAGTCACGAT |
| 28k_292_E07 | TGGAACTACGCCGCAGAAT | ATACGACATACCACCCATCCTT |
| 28k_302_B12 | AGGGATTGTCGGAAGATGC | TCAACCACCAGCCTACCAG |
| 28k_302_B01 | GCAAACTGGGTTGTTGAAATG | ATGTCACGGTCGCAGGTAAT |
| *EF1α* | AGACCACCAAGTACTACTGCAC | CCACCAATCTTGTACACATCC |
