# Supplementary material for: Molecular Mechanisms of Fiber Differential Development between G. barbadense and G. hirsutum Revealed by Genetical Genomics
Source: PLoS One. 2012 Jan 11;7(1):e30056. doi: 10.1371/journal.pone.0030056 (PMC3256209; doi:10.1371/journal.pone.0030056)
Supplement: Table S6 — Gene specific primers used in this study for mapping. (DOC) [file pone.0030056.s008.doc]

**Table S6** Gene specific primers used in this study for mapping

| **Array ID** | **Forward Primer 5'- 3'** | **Reverse Primer 5'- 3'** |
| --- | --- | --- |
| 28k_273_C12 | CTGGGGCTTCTGAGTCCTGT | GGAGTAGTCTGTAGTGGCGAAG |
| 28k_179_E05 | AATGGCAGATGCTTTCGC | TCTTTATCAATGTCAGCCTCCT |
| 28k_179_F12 | ATGAGAGATGGGTTTCACAGAG | GAAAACCTTGACAGCATGCGCC |
| 28k_175_C09 | ATCAAGTAACCGTCATCAGCAC | CCACAACTACCAAAGTCCCAT |
| 28k_187_A03 | CACCCTTTGCCTTTGTCC | TTATGTTGTTTGGCTCCTCCT |
| 28k_189_C03 | GTTGTATGGCGTGCTCGTT | GCTTAGGGATTACCTGTTATGG |
| 28k_176_F01 | AGAAGCCTTTGTGAAGATGCG | CGATTCCTAAGCCAGTTTGTT |
| 28k_267_H07 | ATGGCATCAAACAAAGGAAG | GGTGGCTGTTCCAAGGTTA |
| 28k_275_A03 | TTATTCAAGGNTACGGCTGCTA | TCAAACAAATCCTGTGGCTC |
| 28k_249_G10 | CGTCGTTGCCTGCTTTCA | CATCTATTTTGCCACTCCTGTC |
| 28k_193_H08 | GCATTTCAGTTCACATCGGTC | GGAGCCCAGTCAAGTTATCAG |
| 28k_209_B08 | GATTTCGGAATGGATTAGACAC | ACTGACGCAGCATAGGGAG |
| 28k_211_E10 | GAGCCTGTCAAATGTCCACG | CGAACTCCCTTTCTCCCAC |
| 28k_170_B07 | CTCTGGTCTTTCAGGGGTTG | GGGTAGTATTGGGGCATTG |
| 28k_273_G04 | CTGCTTGCTTGGGTTGGT | CATTGGCATTGAGGGTTCT |
| 28k_214_F11 | CAACCAAATCACTCCCACAT | TCAGGGTCTACTGCTTCCACT |
| 28k_257_D05 | CTCAGTTCAGCCTCCACCA | AAACCCATTATCGTCTTCCTC |
| 28k_211_C10 | AACTCACTATGGTTCATCGTATC | CATCTGGGAGTGGCTGAC |
| 28k_212_H04 | AAAGTTCCCACGCTCTGT | TACCCCATGAAGCAACGG |
| 28k_096_H08 | CAAGTCTCCCTTTACAACC | GATGAAATGAAGAGGCGC |
| 28k_165_H09 | CCGAACGCACCATCAAAG | CAAACGCTAGAAACTACTCGC |
| 28k_200_A04 | CTTTTCTTCTTTTTGTTCAGTGTAT | GGACACTGACGTTATATCCTCTG |
| 28k_263_F02 | TGCTCAACACAGATTTGGTACT | GCTATCGAACTTTGCAGGAG |
